# Home-based nursing interventions improve knowledge of disease and management in patients with heart failure^1^

**DOI:** 10.1590/0104-1169.0144.2523

**Published:** 2015

**Authors:** Karina de Oliveira Azzolin, Dayanna Machado Lemos, Amália de Fátima Lucena, Eneida Rejane Rabelo-Silva

**Affiliations:** 2 PhD, Adjunct Professor, Escola de Enfermagem, Universidade Federal do Rio Grande do Sul, Porto Alegre, RS, Brazil; 3 Master's student, Universidade Federal do Rio Grande do Sul, Porto Alegre, RS, Brazil. RN, Hospital de Clínicas de Porto Alegre, Porto Alegre, RS, Brazil; 4 PhD, Associate Professor, Universidade Federal do Rio Grande do Sul, Porto Alegre, RS, Brazil

**Keywords:** Nursing Process/Classification, Home Visit, Heart Failure, Outcome Assessment (Health Care)/Classification

## Abstract

**OBJECTIVE:**

to assess patient knowledge of heart failure by home-based measurement of two NOC Nursing Outcomes over a six-month period and correlate mean outcome indicator scores with mean scores of a heart failure Knowledge Questionnaire.

**METHODS:**

in this before-and-after study, patients with heart failure received four home visits over a six-month period after hospital discharge. At each home visit, nursing interventions were implemented, NOC outcomes were assessed, and the Knowledge Questionnaire was administered.

**RESULTS:**

overall, 23 patients received home visits. Mean indicator scores for the outcome Knowledge: Medication were 2.27±0.14 at home visit 1 and 3.55±0.16 at home visit 4 (P<0.001); and, for the outcome Knowledge: Treatment Regimen, 2.33±0.13 at home visit 1 and 3.59±0.14 at home visit 4 (P<0.001). The correlation between the Knowledge Questionnaire and the Nursing Outcomes Classification scores was strong at home visit 1 (r=0.7, P<0.01), but weak and non significant at visit 4.

**CONCLUSION:**

the results show improved patient knowledge of heart failure and a strong correlation between Nursing Outcomes Classification indicator scores and Knowledge Questionnaire scores. The NOC Nursing Outcomes proved effective as knowledge assessment measures when compared with the validated instrument.

## Introduction

Patients' knowledge deficits regarding heart failure (HF), its management, and self-care measures have been considered predictors of clinical instability and consequent readmission^(^ 1 ^-^ 3 ^)^. Studies stress that readmission could be prevented in approximately 40-59% of patients with HF by careful discharge planning, proper rehabilitation, identification of potential noncompliance with medications, and instruction of patients and relatives to enable them to early recognize the signs and symptoms of decompensated HF^(^ 4 ^)^. 

Several approaches to the follow-up of these patients have been developed in an attempt to improve knowledge and self-care skills. In addition to follow-up at HF clinics and phone-based monitoring, home visits (HVs) have proved effective in this regard^(^ 5 ^-^ 7 ^)^. However, among these approaches, HVs stand out as a new frontier in the care of chronic patients and permit isolating the effects of nursing interventions from the effects of interventions pertaining to other professions involved in patient care^(^ 8 ^-^ 10 ^)^. 

Within this perspective, the present study sought to assess patient knowledge of HF by home-based measurement of two Nursing Outcomes Classification (NOC) outcomes over a six-month period and measurement of the correlation between mean outcome indicator scores and mean scores on an HF Knowledge Questionnaire (KQ) previously validated for use in Brazil^(^ 11 ^)^. The relevance of this study lies in its use of an intervention protocol based on the Nursing Interventions Classification (NIC) in the measurement of NOC outcomes during home visits, and in the assessment of the correlation between NOC outcomes and a validated patient knowledge evaluation instrument. 

## Methods

### Design

This was a before-and-after study, a sub-analysis from a study that included patients with a clinical diagnosis of HF who were recruited from two public teaching hospitals in Southern Brazil from April 2010 through March 2011^(^ 12 ^)^.

### Sample

The study convenience sample comprised adult patients with systolic HF who were admitted for acute decompensation. Patients with barriers to effective communication, those who lived more than 20 km away from the institution which they were admitted to, and those who did not have a telephone number for later contact were excluded. 

Sample size was calculated in the software WinPepi v.10.5. In that study, a 0.5-point difference between two consecutive NOC evaluations was used to indicate outcome improvement, based on a pilot previously performed in a sample of ten patients. In order to achieve a confidence interval of 90%, an alpha of 1%, a 0.7 standard deviation for the scores and an estimated correlation of 0.5 between the first and fourth visit, and considering a 20% loss, a minimum sample size of 17 patients was calculated.

### Data collection

Data collection took place in the course of four home visits during a six-month period, at days 10, 30, 60, and 120 after hospital discharge. During the first visit (HV1), each patient was interviewed for collection of data on living conditions and health status and underwent a thorough clinical assessment, consisting of a complete physical examination to support the establishment of the nursing diagnoses (NDs) *Readiness for Enhanced Therapeutic Regimen Management *and *Ineffective Self-Health Management*. This was followed by the measurement of the nursing outcomes *Knowledge: Medication* (NOC 1), which consists of five indicators (*Recognition of need to inform health provider of all medications being taken, Statement of correct medication name, Description of actions of medication, Description of side effects of medication,* and *Description of correct administration of medication*), and *Knowledge: Therapeutic Regimen* (NOC 2), with seven indicators (*Description of disease process, Description of rationale for treatment regimen, Description of self-care responsibilities for emergency situations, Description of expected effects of treatment, Description of prescribed diet, Description of prescribed medication, *and *Description of prescribed activity*). 

The indicators were assessed by means of a five-point Likert scale, where "1" is the worst possible score and "5" the best possible score. Operational definitions were developed for each NOC indicator, taking into account each level on the Likert scale, with a view to the standardization of the indicator application. The KQ, which has been validated for use in Brazil, was used as the gold-standard for comparison in this study, and patient scores on this instrument were tested for correlation with NOC outcome indicator scores^(^ 11 ^)^. 

The KQ includes items on diet, fluids and weight, general HF information, medication, physical activity, measures that improve HF, and reasons for readmission, totaling 14 multiple-choice items with four possible alternatives each. The sum of correct answers ranges from 0 to 100; the higher the score, the greater the patient's knowledge of HF^(^ 11 ^)^. Both the NOC outcomes and the KQ were administered at HV1 to establish a baseline for comparison and then re-administered at each subsequent visit (HV2, HV3, and HV4).

After baseline assessment at HV1, the following nursing interventions described under the Patient Education class, Behavioral domain, of the NIC were implemented:* Self-Modification Assistance, Behavior Modification, Health Education, Teaching: Prescribed Medication, *and *Teaching: Disease Process*. These procedures were carried out by a specialist HF nurse. All interventions were reinforced at each subsequent visit (HV2, HV3, and HV4).

The NDs, interventions and outcomes implemented were established by expert consensus^(^ 13 ^)^ and are constituent parts of the study protocol. 

### Ethical considerations

This project received approval from the relevant institutional Research Ethics Committee under protocol number 100055. All patients included in the study had read and signed a free and informed consent form prior to participation.

### Data analysis

Data analysis was carried out using the Statistical Package for Social Sciences (SPSS) version 18.0. Continuous variables were described by calculating the mean and standard deviation (for variables with a normal distribution) or median and interquartile range (for variables that did not present a normal distribution). Categorical variables were expressed as absolute and relative frequencies. 

A cutoff point of 70% of right answers was considered satisfactory for the KQ. For the NOC outcomes, the scores for each indicator (1 to 5) were added and averaged for each outcome. A score of 3 (moderate) to 5 (substantial) for each indicator was considered optimal. Comparison of the average for the outcomes and NOC indicators was carried out using generalized estimating equations (GEE); results were considered statistically significant if the two-tailed p-value was < 0.05. The Pearson correlation coefficient was used to assess the strength of association between the NOC 1 and NOC 2 outcomes and the KQ scores. Towards this end, results for the two NOC outcomes were pooled to generate a single variable.

## Results

During the study period, 532 patients were potentially eligible. Of these, 10% were invited to participate in this study through a convenience sample, but 5% refused to participate. The others were excluded for other reasons (difficulty in communicating, no telephone number available or lived for over 20 km far from the institution).

Overall, 23 patients received four home visits. During the follow-up, two patients did not receive the HV 3, as one of them died and another was travelling; three did not receive HV 4, two of whom died and one moved to anothercity, totaling 87 HV performed.

Most patients were male; the mean age was 63 years, and the mean time elapsed since diagnosis of HF was >3 years (Table 1). 

**Table 1 -:** Socio-demographic and clinical profile of patients with heart failure. Porto Alegre, RS, Brazil, 2010-2011

Variable		n=23
Age, years*		63.3 (±11)
Sex, male^†^		15 (65)
Occupational status, retired^†^		14 (61)
Marital status, married/cohabiting^†^		16 (70)
Living arrangement, cohabiting^†^		11 (48)
Household income, up to 3x minimum wage^†^		16 (70)
Educational attainment, years^*^		7 (±3)
Ethnicity/skin color, white^†^		17 (74)
Etiology of Heart Failure, idiopathic^†^		10(43,5)
Functional class at baseline, New York Heart Association III^†^		7 (30)
Left ventricular ejection fraction (%)*		30 (±8)
Duration of Heart Failure, months^‡^		36 (1-480)
Admissions in last year^†^		7 (30)
Comorbidities		
	Hypertension^†^	14 (61)
	Diabetes mellitus^†^	10( 43)

* Mean ± standard deviation;

† n (%); ‡median (interquartile range).

At HV1, the mean (SD) KQ score was 69.1±19.1; 52.1% of patients (n=12) did not reach the 70% cutoff. At the end of the study period, the mean KQ score was 87.4±8.70.

The mean of all indicators for the *Knowledge: Medication *outcome (NOC 1) at HV1 was 2.28 points (limited). At HV4, the mean score was 3.55±0.16 (moderate). The mean score for the *Knowledge: Treatment Regimen *outcome (NOC 2) was 2.33 (limited) at HV1, versus 3.59 (moderate) at the end of the six-month study period (Table 2).

**Table 2 -:** Mean scores for each indicator of the nursing outcomes *Knowledge: Medication* (NOC1) and *Knowledge: Treatment Regimen* (NOC2). Porto Alegre, RS, Brazil, 2010-2011.

Nursing Outcomes/Indicators		HV1* (n = 23)	HV2* (n = 23)	HV3* (n = 21)	HV4*^†^ (n = 20)
		Mean (Standard Error)			
Knowledge: Medication (NOC 1)^ ‡^		2.28 (0.14)	2.67 (0.14)	3.00 (0.17)	3.55 (0.16)
	Recognition of need to inform health provider of all medications being taken	2.57 (0.18)	2.96 (0.17)	3.33 (0.19)	3.70 (0.18)
	Description of correct administration of medication	2.43 (0.19)	2.91 (0.14)	3.19 (0.19)	3.85 (0.18)
	Statement of correct medication name	2.35 (0.19)	2.70 (0.20)	2.95 (0.19)	3.55 (0.22)
	Description of actions of medication	2.13 (0.21)	2.61 (0.17)	2.76 (0.19)	3.40 (0.17)
	Description of side effects of medication	1.91 (0.16)	2.13 (0.19)	2.76 (0.22)	3.25 (0.19)
Knowledge: Treatment Regimen (NOC 2)^‡^		2.33 (0.14)	2.75 (0.12)	3.13 (0.14)	3.59 (0.14)
	Description of prescribed diet	2.64 (0.18)	3.22 (0.14)	3.62 (0.15)	3.80 (0.14)
	Description of prescribed medication	2.52 (0.16)	2.83 (0.14)	3.24 (0.17)	3.65 (0.20)
	Description of prescribed activity	2.52 (0.18)	3.09 (0.12)	3.33 (0.14)	3.75 (0.12)
	Description of self-care responsibilities for emergency situations	2.26 (0.17)	2.61 (0.19)	2.95 (0.22)	3.50 (0.21)
	Description of expected effects of treatment	2.22 (0.19)	2.52 (0.16)	2.86 (0.20)	3.45 (0.15)
	Description of disease process	2.22 (0.20)	2.57 (0.15)	3.10 (0.20)	3.50 (0.18)
	Description of rationale for treatment regimen	2.09 (0.20)	2.43 (0.18)	2.81 (0.20)	3.50 (0.18)

* Home Visit

† Three patients had been lost to follow-up by HV4

‡ Nursing Outcomes Classification

**Figure 1 -:**
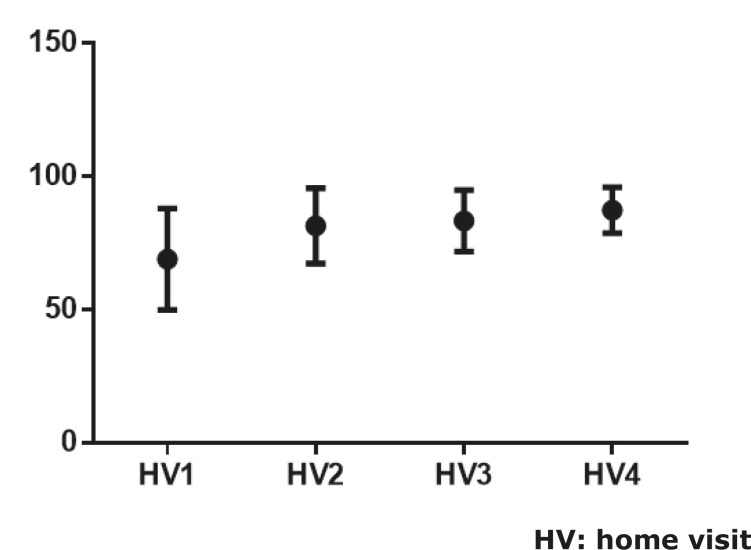
Disease Knowledge and Self-Care Questionnaire scores of patients with heart failure at each study visit (P<0.001).

The mean for the Disease Knowledge and Self-Care Questionnaire scores at HV1 was 69.1±19.1. At HV4, the mean score was 87.4±8.70 (Figure 1).

**Figure 2 -:**
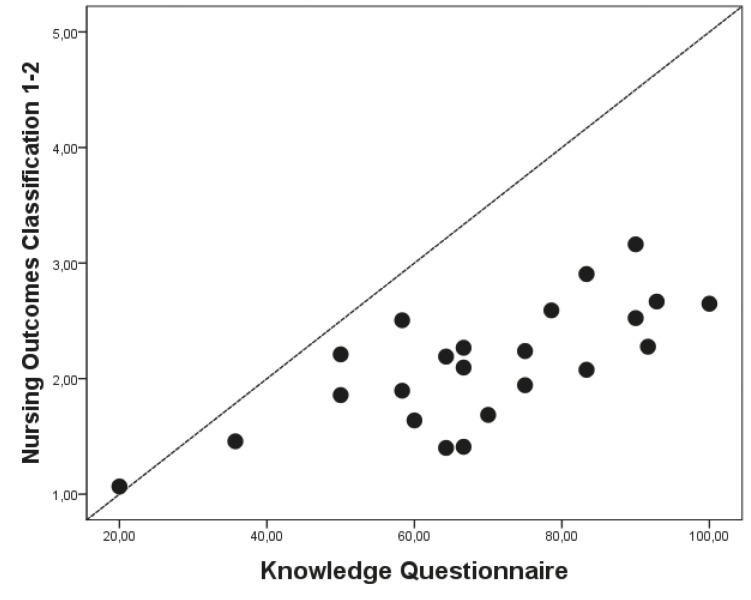
Correlation between NOC outcome indicator scores and Knowledge Questionnaire scores at home visit 1 (r=0.7, P<0.001).

Assessment of the correlation between NOC outcomes and KQ scores was carried out to ascertain the utility of the NOC outcomes as assessment metrics. Therefore, a single variable was constructed by pooling the mean scores of the NOC1 and NOC2 outcomes and tested for correlation with KQ scores. At HV1, this correlation was strong (Figure 2).

There was no correlation between KQ scores and NOC outcome scores (1 and 2) at HV4, as the mean scores for both had increased significantly (r=0.3, P=0.084).

## Discussion

This was the first study to conduct a home-based assessment of the knowledge of patients with HF by means of NOC outcomes and the first to correlate these outcomes with a validated instrument for purposes of knowledge assessment in this patient population. Both instruments revealed a significant improvement in patient knowledge after home-based nursing interventions.

At baseline, both instruments showed that patients had a deficit in knowledge of HF and self-care measures. Several causes may be involved in this deficit. These include healthcare providers who do not have enough time to give patients appropriate guidance at several points in the care process (during scheduled visits, during treatment in the emergency department setting, during admission, and at hospital discharge); poor training of hospital staff in patient and family education; and unavailability or inaccessibility of effective management in the primary care setting. 

A study designed to identify the learning needs of patients with heart disease found that 95% of participants had deficient knowledge of disease and treatment. The worst scores were obtained on items concerning signs and symptoms of complications: 53.1% of patients provided completely wrong answers or were unable to answer at all^(^ 14 ^)^.

These findings highlight the importance of assessment of nursing interventions in chronically ill patients, particularly in populations with a high rate of readmission (such as patients with HF).

In the present study, we used the NOC outcome *Knowledge: Treatment Regimen*. This outcome was previously assessed in a study of hospitalized patients with chronic disease (including HF) divided into three groups: group 1 (n = 91) received training for the post-discharge period; group 2 (n = 103) received both discharge training and a telephone-based intervention; and group 3 (n = 52) received the two aforementioned interventions and a home visit after hospital discharge. The nursing interventions implemented in this study were *Discharge Planning, Caregiver Support, Health Education, Teaching: Disease Process, *and *Teaching: Individual. *Diverging from our findings, this study identified a significant improvement in the *Knowledge: Treatment Regimen *outcome in group 2 alone^(^ 15 ^)^, although only one home visit was performed. 

Another study that assessed the implementation of nursing interventions over the course of five home visits in a sample of chronically ill patients found a significant improvement by 1.1 points in indicator scores for the NOC outcomes assessed, with a final result corresponding to moderate knowledge of treatment^(^ 16 ^)^. These findings are similar to those reported herein. In this context, the duration of follow-up and greater number of visits may have influenced improvement in *Knowledge: Treatment Regimen *indicators. The interventions implemented were *Teaching: Disease Process*, *Teaching: Prescribed Medications, Teaching: Prescribed Diet, Exercise Promotion *and *Behavior Modification.*

Better knowledge of one's disease and its management enables towards proper self-care. A Colombian randomized clinical trial of the effectiveness of a nurse-led education program showed improvement in self-care behaviors among patients with HF. In the intervention group, 66.0% of patients exhibited an improvement in self-care by at least 20%, versus 26.6% of controls (p<0.001)^(^ 17 ^)^.

A randomized clinical trial that used KQ to evaluate a one-hour-long heart failure education program by a nurse educator demonstrated significantly higher total score increases in the intervention group (*n* = 113) compared to patients receiving the standard discharge process (*n* = 114) (median, IQR 1.0 to 4 versus 0.2 to 2, p = 0.007). In addition, significantly lower KQ scores were found in patients who experienced death or rehospitalization in six months (10.7 to 12 versus 11.8 to 13, p = 0.002)^(^ 18 ^)^.

Mean indicator scores for the outcome *Knowledge: Medication *also increased significantly from HV1 to HV4, corresponding to an improvement from limited knowledge to moderate knowledge (P < 0.001). All indicators improved from HV1 to HV4. Similar findings for this outcome were found in a previous study of patients with HF treated by nurses in a day hospital setting (P < 0.001 at 5th assessment)^(^ 19 ^)^. 

The KQ instrument was used in this study because it has already been validated for assessment of knowledge in patients with HF, unlike NOC outcomes, which had never been clinically validated for this purpose. Another study that employed the KQ in patients with HF found that 40% of patients who had satisfactory knowledge of their condition were treatment-adherent, whereas only 13% of non-adherent patients had such knowledge^(^ 1 ^)^. 

The increase in mean scores on both scales shows that NOC outcomes are appropriate to assess knowledge in patients with HF. 

## Conclusion

The findings of this study revealed a significant improvement in NOC outcomes that assess knowledge of disease and its management among patients with HF after the implementation of nursing interventions over the course of four home visits. Comparison of these results with KQ findings showed that patients who achieved the best NOC scores also had the highest mean KQ scores. Therefore, we conclude that patient knowledge of HF and its management was enhanced by the study intervention, with potential positive implications for their health status. Finally, we conclude that NOC outcomes are effective tools to assess the impact of educational nursing interventions, as their performance was comparable to that of a clinically validated questionnaire.
